# Spanning the gap: unraveling RSC dynamics in vivo

**DOI:** 10.1007/s00294-020-01144-1

**Published:** 2021-01-23

**Authors:** Heinz Neumann, Bryan J. Wilkins

**Affiliations:** 1 Department of Structural Biochemistry, Max-Planck-Institute of Molecular Physiology, Otto-Hahn-Strasse 11, 44227 Dortmund, Germany; 2 Department of Chemical Engineering and Biotechnology, University of Applied Sciences Darmstadt, Stephanstrasse 7, 64295 Darmstadt, Germany; 3 Department of Chemistry and Biochemistry, Manhattan College, 4513 Manhattan College Parkway, Bronx, NY 10471 USA

**Keywords:** Genetic code expansion, Unnatural amino acids, Photo-crosslinking, Chromatin remodelling, RSC, Sumoylation, Lysine acetylation

## Abstract

Multiple reports over the past 2 years have provided the first complete structural analyses for the essential yeast chromatin remodeler, RSC, providing elaborate molecular details for its engagement with the nucleosome. However, there still remain gaps in resolution, particularly within the many RSC subunits that harbor histone binding domains.

Solving contacts at these interfaces is crucial because they are regulated by posttranslational modifications that control remodeler binding modes and function. Modifications are dynamic in nature often corresponding to transcriptional activation states and cell cycle stage, highlighting not only a need for enriched spatial resolution but also temporal understanding of remodeler engagement with the nucleosome. Our recent work sheds light on some of those gaps by exploring the binding interface between the RSC catalytic motor protein, Sth1, and the nucleosome, in the living nucleus. Using genetically encoded photo-activatable amino acids incorporated into histones of living yeast we are able to monitor the nucleosomal binding of RSC, emphasizing the regulatory roles of histone modifications in a spatiotemporal manner. We observe that RSC prefers to bind H2B SUMOylated nucleosomes in vivo and interacts with neighboring nucleosomes via H3K14ac. Additionally, we establish that RSC is constitutively bound to the nucleosome and is not ejected during mitotic chromatin compaction but alters its binding mode as it progresses through the cell cycle. Our data offer a renewed perspective on RSC mechanics under true physiological conditions.

Advances in cryoelectron microscopy have led to several high-resolution structures of nucleosome-bound chromatin remodeler complexes, within the past few years (Ayala et al. 2018; Eustermann et al. 2018; Ye et al. 2019; Wagner 2020; Han 2020). While these reports have detailed, quite beautifully, many of the contacts that the complexes make with the nucleosome, they are nevertheless incomplete, leaving unresolved, several auxiliary subunit-to-nucleosome interactions, particularly histone binding interfaces. Remodelers from all families possess histone binding domains within their numerous subunits influencing overall activity. The biological relevance of defined contacts remains to be seen and there are gaps in our comprehensive map of remodeler histone binding domains and their in vivo significance.

Chromatin remodelers control cellular processes such as transcription, replication and differentiation as well as having essential roles in the DNA damage response pathways (Simone 2006; Lans et al. 2012; Tsabar and Haber 2013; Smerdon 1991). Unraveling the mechanistic details of each chromatin remodeler has historically proven to be a daunting task, as remodeler complexes are large, multimeric protein structures that are involved in elaborate protein–protein and protein-DNA stabilizing interactions. Histone posttranslational modifications (PTMs) are well-known gatekeepers of remodeler function, facilitating trans-acting sequestration and access to DNA sequences essential to downstream biochemical processing.

Open questions still remain regarding details on how histone modifications regulate remodelers and how the mechanistic function of the modifications influence translocation and recruitment events. How are remodeler complexes assembled and sequestered to the nucleosome? Histone modifications have long been thought to control these events, but it is yet to be seen how recruitment is facilitated, under physiological conditions. Importantly, if established PTM models of recruitment are challenged, what function do these essential modifications serve during remodeling events? While these questions are important for the entirety of the superfamily of remodeler proteins there has been a rather substantial amount of recent work addressing the structure of the *Saccharomyces cerevisiae* SWI/SNF subfamily remodeler complex, RSC. RSC is essential in yeast and contains 17 subunits, where its catalytic activity is dependent upon the ATPase, Sth1, translocating DNA in approximately 1–2 bp incremental steps (Du et al. 1998; Cairns et al. 1996; Harada et al. 2016; Zhang et al. 2006). How the entire ensemble of RSC subunits is assembled and associated with the nucleosome is only now coming into view.

Histone hyperacetylation is a defining marker of active chromatin and the SWI/SNF family of remodelers are established regulators of transcription. (Parnell et al. 2008) Both, SWI/SNF and RSC possess bromodomains that read acetylated lysines and have each been functionally linked to acetylation requirements for activation (Agalioti et al. 2002; Dilworth et al. 2000; Chatterjee et al. 2011). Accordingly, it is not surprising that RSC recruitment has been correlated to direct contacts made with the H3 tail at acetylated lysine 14 (H3 K14) through the Rsc4 subunit (Kasten et al. 2004; Chatterjee et al. 2011; VanDemark et al. 2007). RSC has been shown to have a greater affinity for H3 K14 acetylated nucleosomes and that docking at this position stabilizes the nucleosomal-remodeler complex, promoting chromatin reorganization (Duan and Smerdon 2014). Accepted models for RSC engagement with the nucleosome state that initial recruitment of the remodeler to the site of translocation is reliant on H3 acetylation marks. Here, it is important to emphasize that current structures cannot resolve the contacts between Rsc4 and histone H3, as previously reported from in vitro studies (Chatterjee et al. 2011). Significantly, the bromodomains of Sth1, Rsc4, Rsc1/2, and Rsc58 are all missing contact resolution with the nucleosome. Even elegant structural studies offer an incomplete picture of the structure and functional dynamics of the complex. This highlights the importance of alternative and complementing methods for determining nucleosomal contacts with remodeler complexes.

A growing number of recent reports have revealed the versatility of an in vivo approach to protein–protein interaction studies utilizing UV-activatable unnatural amino acid (UAA) crosslinkers (Coin et al. 2013; Wagner 2020; Shah 2020). These UAAs are incorporated into proteins of living cells through the heterologous expression of an orthogonal aminoacyl-tRNA synthetase (aaRS)/tRNA_CUA_ pair in the host organism (Fig. 1). The aaRS of this pair is designed to load the desired UAA onto its cognate tRNA, which in turn is designed to direct the incorporation of this UAA in response to amber codons. Hence, by replacing a sense codon in the gene of interest with an amber codon, proteins are obtained with the UAA at the corresponding residue (Liu & Schultz 2010; Neumann 2012; Neumann et al. 2018). These techniques were established as a viable tool for chromatin exploration, in the living nucleus, via the expression of crosslinking histones harboring the unnatural amino acid, *p*-benzoylphenylalanine (pBPA), allowing for the distribution of crosslinking probes throughout the native chromatin landscape (Fig. 1) (Wilkins et al. 2014; Hoffmann and Neumann 2015). Upon whole-cell irradiation at  ~ 365 nm, pBPA is activated to a radical intermediate that can form covalent bonds with neighboring proteins through a hydrogen abstraction mechanism (Dorman and Prestwich 1994). The covalently crosslinked products can be assessed by Western blotting, enabling for the identification and spatiotemporal quantification of molecular contacts of histone proteins under native conditions.

**Fig. 1:**
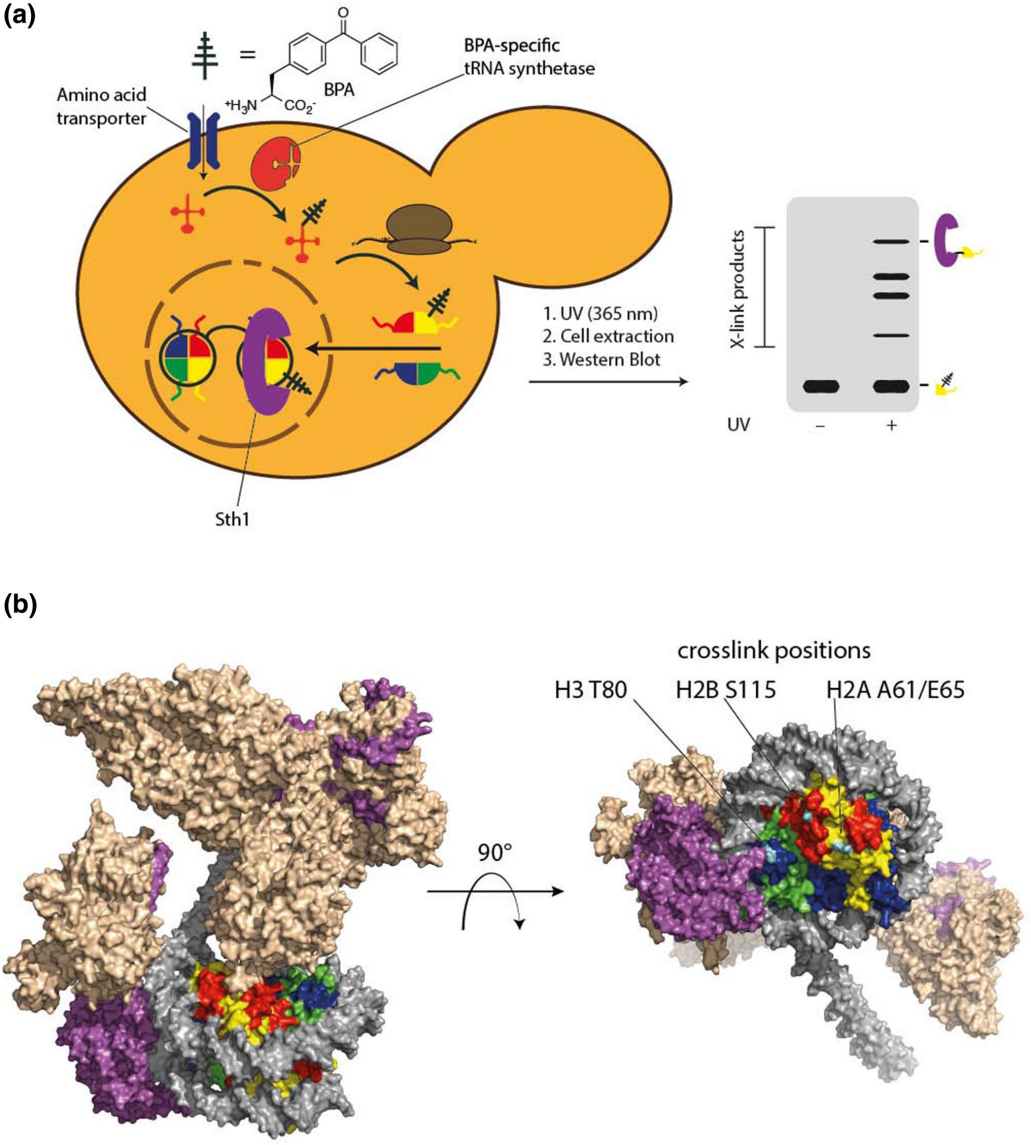
**a** Genetic code expansion facilitates the incorporation of photo-activatable amino acids in histone proteins in living yeast. The crosslinker-containing histone is incorporated into the chromatin landscape and forms covalent adducts with binding proteins, such as the Sth1 subunit of RSC, upon irradiation. Adducts are subsequently detected by Western blot. **b** Crosslink-products formed between histones and Sth1 are mapped onto the structure of the nucleosome-bound RSC complex. These residues are likely in contact with the SnAC domain of Sth1, which is not resolved in the structure (image generated using pdb-file 6TDA and Chimera)

This approach is extremely powerful for monitoring individual protein–protein interactions in living cells. Contact surfaces between proteins can be mapped by moving the position of the crosslinker UAA across the interface (Jain et al. 2020; Hoffmann and Neumann 2015). The crosslinking reaction is highly reproducible, facilitating the quantification of the influence of PTMs or the cell cycle stage on these interactions (Wilkins et al. 2014; Kruitwagen et al. 2015; Jain et al. 2020). Unfortunately, the throughput of the approach is fairly low and the experiments technically demanding. The introduction of the amber suppressor system impacts the general physiology of the cell, for example, by suppression of natural amber codons, and care must be taken not to disturb the process under investigation. However, since the insights from this approach are hard to obtain by any other method, the effort is well spent.

Recent work in our labs has exploited this crosslinking technique to provide further insight into histone contacts with the RSC motor domain, in vivo (Jain et al. 2020). We characterized several unique Sth1 crosslinks with the histone H2A acidic patch as well as with the histone H3 core and tail, offering physiological evidence for PTM and cell cycle regulation of RSC binding to the nucleosome (see Fig. 1 for an overview of crosslinking assays). A point of particular interest is the fact that our data does not support a sequestering mechanism, in vivo, that relies on H3 tail acetylation, contrary to prevalent remodeler-nucleosome recruitment models (Patel et al. 2019; VanDemark et al. 2007). RSC is positioned at the nucleosome prior to acetylation events and we suggest that the modification, rather than signaling RSC engagement, controls motor domain function by mediating DNA unspooling, particularly of neighboring nucleosomes. Additionally, we observed that RSC binding to histone H2B was controlled by SUMOylation, in vivo, however, the modification only appeared to have a modest influence on RSC affinity for nucleosomes, in vitro. Remodeling assays, in the presence of SUMOylated nucleosomes, did not reveal a significant increase in activity but, interestingly, we observed a negative influence on nucleosome ejection. It is yet unclear how SUMOylation mediates RSC activity and further work will be needed to address how the role of the modification influences RSC dynamics. Collectively, our data enhances current RSC-nucleosome structural and functional mechanistic models, under physiological conditions.

We used quantitative cross-linking assays to reveal that Sth1 interacts with histones H3 (S22 and K56) and H2A (A61) with a constant binding efficiency, regardless of cell cycle or PTM mutations. This denotes that the remodeler-nucleosome interaction is not muted as a result of mitotic chromosomal condensation and that RSC appears to be constitutively bound, prepositioning the remodeler at specified nucleosomes. Conversely, we observed additional highly efficient crosslinking positions from histones H3 (T80) and H2B (T51) that displayed reciprocal binding efficiencies (T80 maximized in mitosis and T51 in interphase) indicating there are clear variations in the binding mode of the protein at these positions dependent on the chromosomal organization. Additionally, multiple positions of crosslinking from the histone H3 tail (T6 and T11) were more dynamic due to the fact that they were readily controlled by acetylation of H3 at lysine 14. In the presence of H3 K14 acetylation, crosslinking to Sth1 was observed from histone H3 T6 and H3 T11 residues. In contrast, when a H3 K14A mutant was explored, Sth1 binding to the H3 tail could no longer be detected. Due to the long-standing notion that acetylation sequestered Rsc4 it is quite intriguing that Sth1 was also controlled by the same modification (Kasten et al. 2004; VanDemark et al. 2007). Recent work supports this point, revealing structural analysis of the N-terminal tail of histone H3 bound to the C-terminal Sth1 bromodomain (Chen et al. 2020). Sth1 binding was dependent upon acetylation of lysine 14 and further suggested that Sth1 is the most significant reader of this modification, even more so than Rsc4. Collectively, current data suggest that H3 K14 acetylation is a key regulator of RSC function, beyond a simple sequestering signal.

Sth1 nucleosomal crosslinking was further characterized in the absence of H3 K14 acetylation and we verified that histone H3 S22 and H2A A61 crosslinks remained constitutive, and were not influenced by the modification. These results correlate well with the fact that H3 S22 binding efficiency was not altered across the cell cycle and that this position must act as a docking site for the stabilization of the protein at the nucleosomal interface. This site being located spatially adjacent to H3 T6 and T11 suggests that the complex is primed for rapid action at the H3 tail upon acetylation events.

RSC function in the eviction of H2A-H2B dimers implicates contacts to the nucleosome acidic patch. Interestingly, however, each of the recent RSC cryo-EM reports verified an acidic patch association, though, not with Sth1, but with the SRM (Substrate Recognition Module) protein Sfh1. In fact, the structure reported by Ye et al. does not resolve Sth1 binding to the H3 tail, or the acidic patch (Ye et al. 2019). Additionally,  ~ 300 residues of the Sth1 C-terminal domain, where the bromodomain resides, are missing. Our data, in combination with structural determinations, supports a model where RSC engages the nucleosome on both faces, with Sth1 binding one acidic patch, most likely via its SnAC domain, and Sfh1 binding the other face, involving a nucleosomal sandwiching between the motor and SRM modules (Fig. 1). The SnAC domain sits at the terminal end of the RSC-nucleosome complex and possibly stabilizes the nucleosome during translocation, while Sfh1 works to hold the opposing face, stabilizing the nucleosome during the motor/ARP/hinge control of DNA driving toward the dyad exit. This dual face binding is further reinforced by the fact that the Sth1 bromodomain resides subsequent to the SnAC, and Rsc4 within the same module as Sfh1. This positions each of the H3 acetylation binding domains at opposite surfaces of the nucleosomal disc, reconciling data with the latest structures.

Current models suggest a multi-step mechanism for RSC binding the nucleosome that is initiated by recruitment to acetylated H3 tails followed by binding of the SRM (Patel et al. 2019). Our work highlights that H3 K14 acetylation is not a sequestering mechanism, however the loss of acetylation negatively influences the binding of Sth1 to the most terminal region of the H3 tail (see Fig. 2. for proposed PTM effects on remodeler activity). We propose a model of RSC nucleosomal engagement where the H2A acidic patch, most likely binding the Sth1 SnAC, and the H3 tail, with S22 positioned adjacent to the bromodomain, act as anchors for RSC’s catalytic subunit on the nucleosomal core. The RSC complex remains in contact with the nucleosome at all times and the remodeler is positioned in a “loaded” stage ready to “fire” toward the H3 N-terminal portion of the tail upon acetylation. Binding of the bromodomain to the acetylated H3 tail likely communicates activation to the motor domain through conformational changes and interaction with the ARP (Actin-Related Protein) module of RSC. ARP stabilizes the Sth1, HSA helix, which is connected to the ATPase motor domain through a short flexible hinge region (Wagner 2020; Ye et al. 2019). Translocation events are likely regulated through allosteric effects due to acetylation activated repositioning of the ARP module which influences localized changes through the hinge and post-HSA regions, ultimately acting as a key regulator of translocation events.

**Fig. 2:**
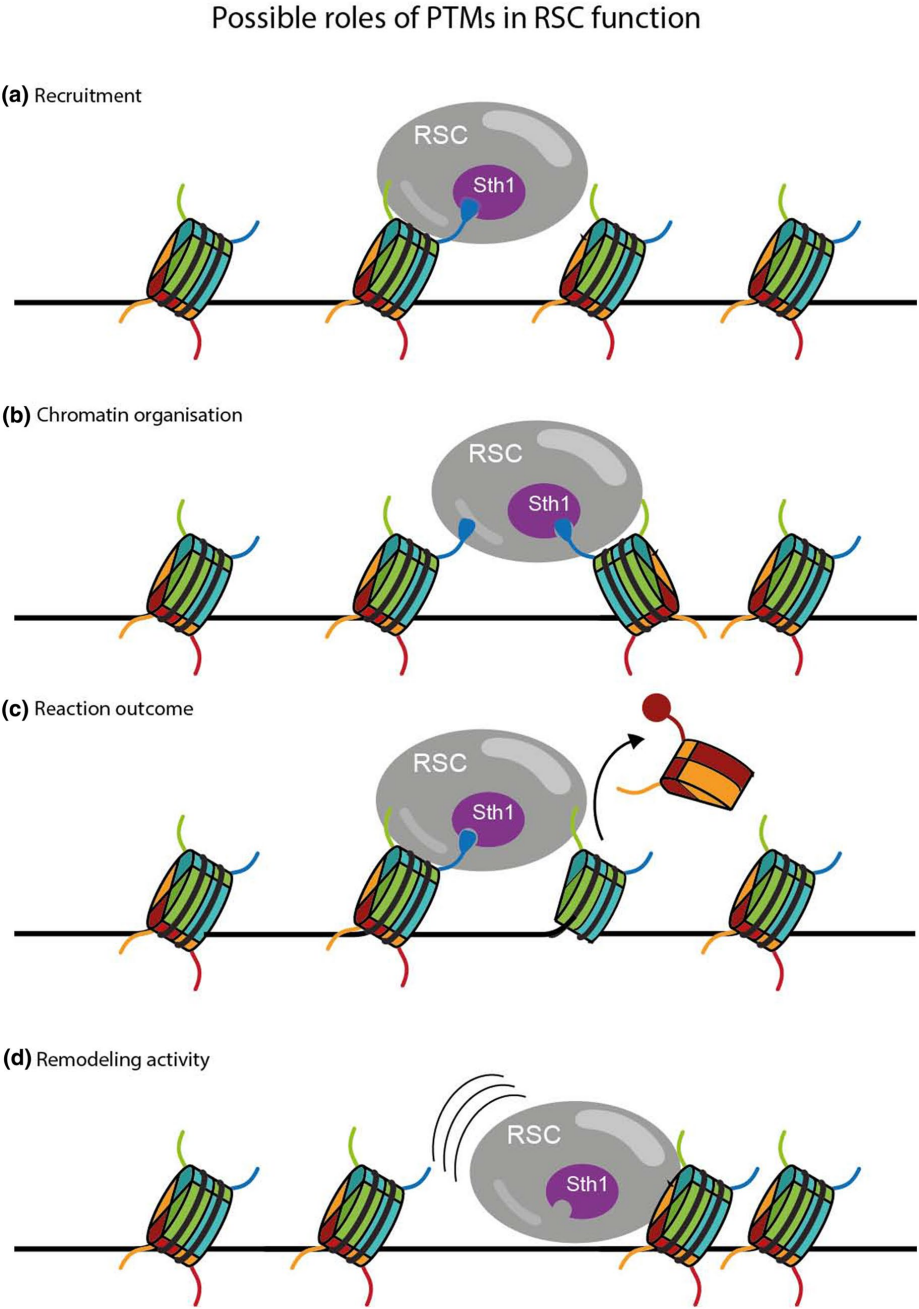
Various ways in which PTMs can affect chromatin remodelers. **a** PTMs, such as H3K14ac, may serve as recruitment signals to direct remodelers to specific genomic loci. **b** Simultaneous binding of multiple histone PTMs might direct the positioning of nucleosomes, e.g. at promoters. RSC recruitment occurs prior to H3 tail acetylation. Rather than a sequestering signal, acetylation stimulates internucleosomal contacts with Sth1, communicating that the loading state has been achieved. **c** The presence of particular PTMs, such as H2B SUMOylation, on substrate nucleosomes may modulate the outcome of the remodeling reaction, e.g. nucleosome repositioning versus eviction. **d** Presence (or absence) of certain PTMs may regulate the translocation speed of the remodeler

Skiniotis et al. (2007) have previously reported a non-recruitment role for H3 tail acetylation for RSC. They observed a two-lobed RSC structure where the lower lobe is mobile and resides in either an open (tilted away from the top lobe) or closed (sitting against the upper lobe) position. RSC nucleosomal binding can only be accommodated in the open state. Acetylation stimulates association of the lower lobe, around the nucleosome, and into close proximity with the upper lobe, stabilizing the RSC closed state. This data supports an alternative mechanism for H3 K14 acetylation that is not working to sequester RSC to the nucleosome but rather communicating, through structural changes in binding, that RSC has acquired the prerequisite loading state for translocation. The lower lobe of their structure would be consistent with the SnAC and bromodomains of Sth1, while the upper lobe would harbor the SRM, connecting each lobe through the HSA and ARP modules. This implicates Sth1 binding at acetylated H3 K14 as the locking mechanism and signal for translocation, which is in significant correlation with our most recent work. Initial recruitment of RSC occurs upstream of acetylation events and the enzyme is conceivably prepositioned at specific nucleosomes, controlled by transcription regulators, DNA sequences, and other mechanistic actions.

Work on the mammalian SWI/SNF homologs, BAF and PBAF, have identified stable complex intermediates, hinting at the ordering of remodeller organization.(Mashtalir et al. 2018) It is likely that the SRM is the first module to assemble followed by subsequent binding to Sth1, and then the recruitment of the ARP module. It is still unclear whether the remodeller is loaded at each face of the nucleosome simultaneously or if engagement is established through Sth1 binding. It is plausible that the motor domain makes initial contacts with the nucleosome, stabilizing interactions at the H3 tail and H2A-H2B acidic patch. Once Sth1 is positioned, the SRM could then engage the opposing acidic patch, via Sfh1, providing bimodal stabilization, while concurrently associating with Sth1 N-terminal domain via the Rsc numbered subunits. The ARP module is a regulator of translocation, and therefore, reasonably assembled last, perhaps via signaling through the Sth1 bromodomain binding to the acetylated H3 tail and structural changes at the post-HSA and hinge regions.

Histone H3 T80 may also play an important role in the initial binding of Sth1. This contact is maximal in mitosis signifying that it may facilitate an inactive binding configuration for RSC. Perhaps T80 stabilizes an “off” position (open state), postponing comprehensive recruitment of RSC to Sth1 until acetylation is upregulated during interphase. Histone H3 K79 methylation, known to be a transcription activation signal, may contribute an important role during the mechanistic switch between inactive and active RSC states. It will be of great importance to this field to resolve completely the assembly mechanism for RSC at the nucleosome and elucidate the temporal regulation of recruitment signals.

Interestingly, our data suggest that the Sth1 bromodomain acts through inter-nucleosomal contacts (Fig. 2b). There are two proposed models for how DNA translocation occurs, leading to nucleosomal ejection (Clapier et al. 2017). The first proposes that DNA movement and loosening from the histone octamer, upon the nucleosome that RSC is bound, leads to enzymatic access for chaperone assisted removal of the histone octamer. The second model suggests that RSC facilitates ejection of the n + 1 neighboring nucleosome. This is referred to as “sliding-mediated nucleosomal disassembly” and occurs when the translocation continues past the end of the linker where RSC associates and begins to unwind a proximal nucleosome (Boeger et al. 2008). Notably, we highlighted that Sth1 preferably binds across nucleosomes upon H3 K14 acetylation, providing evidence for the second model, in vivo. This mechanism does not displace the nucleosome bound by RSC, evicting only its neighbors, establishing boundaries at promoter nucleosome-free regions.

Lastly, we further highlight the complexity of PTM influences on RSC function with the identification of SUMOylation effects on remodeler binding to the nucleosome (Fig. 2c). Histone SUMOylation has been shown to negatively affect transcription in yeast and is anticorrelated with acetylation activation (Nathan et al. 2006). Our assays suggest that SUMOylation, while not directly affecting catalytic efficiency, does inhibit the ability of RSC to eject nucleosomes, to some extent, in vitro. Multiple reports have implicated SUMOylation as a requirement for deacetylase recruitment and perhaps SUMOylation inhibits RSC-driven eviction of nucleosomes through conformational changes in binding mode to modified H2B, and the establishment of the off-state via deacetylation of the H3 tail (Yang and Sharrocks 2004; Shiio and Eisenman 2003).

While it is clear that histone modifications serve in a regulatory role for RSC function, it is important to note that the remodeler can efficiently facilitate nucleosomal translocation on unmodified reconstituted nucleosomes. H3 tail acetylation enhances RSC activity, in vitro, but is not essential for its function (Harada et al. 2016; Chatterjee et al. 2011). In the absence of acetylated N-terminal H3 peptides, RSC exists in a mixture of open and closed states, as discussed above, where acetylation promotes the closed active state (Skiniotis et al. 2007). In the mixed state (no acetylation marks), RSC appears to possess an inherent basal activity that is not fully reliant on modifications to establish a stable catalytic conformation. Although acetylation of the H3 tail is not essential, the presence of the modification does increase the efficiency of nucleosomal movement by twofold and the *K*_D_ by about eightfold (Chatterjee et al. 2011). A basal function of RSC is also apparent in vivo as the H3 N-terminal tail (and H3 K14A mutation) is not essential in yeast, yet RSC itself is. RSC functions in the absence of acetylation and other defining marks, but the modifications are most certainly required for the full catalytic activity of the complex, particularly under physiological conditions. Each subfamily of remodelers is regulated by the dynamic properties of histones and their various PTMs. Ultimately, the combinatorial presence, or absence, of multiple modifications governs the catalytic efficiency of the remodeler.

To date, there are limited techniques that allow for detailed characterization of chromatin dynamics, in vivo, particularly assays that can report on both structural and mechanistic insights, simultaneously. While structural analysis provides a high-resolution view of the nucleosome-remodeler complex there remain significant gaps at points of essential contact. Solution studies have outlined mechanistic insights yet their relevance, in vivo, is difficult to fully assess. Genetic code expansion with crosslinking amino acids provides some of the much-needed clarity that spans the gap between in vitro versus in vivo understanding of chromatin dynamics. Using this approach, we show that RSC chromatin engagement at the nucleosome is constitutively bound under physiological conditions. Histone H3 acetylation is not a recruitment mechanism but rather a dynamic regulator for catalytic activity. Additionally, RSC has an increased affinity for H2B SUMOylation and is controlled by multiple combinations of histone modifications. In summary, spatiotemporal insights from the work detailed here provide an updated perspective on the biological relevance of histone mediated RSC dynamics.
